# Ametropia and the Cardio-Vascular System

**Published:** 1891-09-05

**Authors:** 


### OPHTHALMOLOGY.

### AMETROPIA AND THE CARDIO-VASCULAR SYSTEM.

In a well-reasoned paper read before the Harveian Society some months ago, Dr. Rayner Batten discusses the various points of connection between the eye and the cardio-vascular system, and contends that hypermetropia and myopia should not be regarded as merely local conditions, but as part of a general state of the vascular system. In support of his theory he first refers to the well-known connection between diseases of the eye and vascular diseases, such as Bright's disease, exophthalmic goitre, and megrim. He then comments on the comparative frequency of optic neuritis in hypermetropes. It is still a moot point whether hypermetropia is a sufficient cause for optic neuritis. Gowers thinks that it is at all events possible. What is also significant is the rarity of optic neuritis in myopic subjects. Dr. Batten says he has never seen such a case himself, nor could he find one mentioned amongst descriptions of cases of optic neuritis. He adds: "I have questioned several well-known ophthalmoscopic observers, both surgical and medical, and have obtained opinions drawn from a vast amount of experience, and so far I have collected three cases only of optic neuritis in which myopia was noted. The universal opinion of those I have asked is, that the association of the two is very rare. Some express doubt as to its occurrence." He thinks that the extreme rarity of optic neuritis in myopes is due, not to the comparative rarity of myopia, but either to a structural difference in the eye or in the body of the myope, probably the latter. The high tension pulse, which Broadbent has noticed as being common in glaucoma, he regards as being really associated with the hypermetropia which generally exists in glaucomatus eyes. This view, of course, does not negative the opinion generally held that glaucoma depends partly on the relatively large size of the lens of the hypermetropic eye, and on the continuous effort of accommodation. It also accords with the fact, especially insisted on by Lawson, that glaucoma is generally preceded by mental worry and anxiety.

In examining a great number of patients he comes to the conclusion that the vascular system is usually characterised in hypermetropia by a rapid excitable pulse, with accentuated first heart sound, in myopia by a slow full pulse. In hypermetropes he finds that instillation of atropine or correction by glasses in only a few cases renders the pulse slower, and that neither produces much effect in myopes. This, therefore, supports the view that it is not the effort of accommodation which by reflex action causes the "heart hurry," but rather that the rapid pulse is due to the same cause as the hypermetropia. In proof of the excitable nature of the "hypermetropic" circulation, he mentions the not infrequent faintness under chloroform produced immediately after tenotomy of the internal rectus (an operation which, of course, is rarely performed except in hypermetropes). We have repeatedly noticed this, but have not noticed fainting occur during similar operations on myopes. Another fact Dr. Batten says he has noticed, namely, that the hypermetrope reaches his full development later than the myope. All young children are hypermetropic (averaging about + 3 D. at birth), and the hypermetrope, as it were, carries some of his youthful characteristics into adult life.

While admitting the force of some of these arguments, we must be careful not to confuse the cause with the effect. The myope of, say, 5 D. is, *ipso facto*, differently affected by his environment, and so it may be that his bodily activity and cardio-vascular mechanism become adapted to his surroundings and mode of life. We fully agree that "myopia is not a healthy adaptation to a studious generation any more than is scoliosis, but it is an unfortunate accompaniment." And it seems to us that the "myopic" circulation must be regarded as the one that is best fitted to compensate for the special defects of the myope, and to fulfil his peculiar requirements. The theory that hypermetropia depends on, or, at any rate, is intimately associated with, a peculiar development of the cardio-vascular system (retarded development), is more easily sustained, for, unlike myopia, it is not known that hypermetropia can be induced or increased by use or abuse of the eyes. On the contrary, as we have said, it tends to diminish, and Macnamara, and many others, in ordering spectacles for hypermetropes, adopt the plan of slightly undercorrecting the error of refraction, so as to let nature feel the need of effecting a cure.

The subject is a very wide one, and perhaps Dr. Batten's theory can hardly be regarded as sufficiently proved, but it opens up a field for investigation, not only as to the possibility of checking the progress of myopia and curing hypermetropia by constitutional treatment, but also as to the respective liability of hypermetropes and myopes to various diseases, especially functional disorders of the circulatory system.

